# Dataset on the prognostic value of cardiac biomarkers used in clinical routine in patients with severe aortic stenosis undergoing valve replacement

**DOI:** 10.1016/j.dib.2020.105111

**Published:** 2020-01-09

**Authors:** Fabian Barbieri, Thomas Senoner, Agne Adukauskaite, Stephan Dobner, Johannes Holfeld, Severin Semsroth, Thomas Lambert, David Zweiker, Thomas Theurl, Peter Rainer, Albrecht Schmidt, Gudrun Feuchtner, Clemens Steinwender, Uta Hoppe, Florian Hintringer, Axel Bauer, Silvana Müller, Michael Grimm, Bernhard Pfeifer, Wolfgang Dichtl

**Affiliations:** a University Clinic of Internal Medicine III, Medical University Innsbruck, Innsbruck, Austria; b Department of Cardiology, Inselspital, Bern University Hospital, University of Bern, Switzerland; c University Clinic of Heart Surgery, Medical University Innsbruck, Innsbruck, Austria; d Department of Cardiology, Kepler University Hospital, Medical Faculty, Johannes Kepler University Linz, Austria; e Department of Internal Medicine, Division of Cardiology, Medical University Graz, Graz, Austria; f University Clinic of Internal Medicine II, Paracelsus Medical University, Salzburg, Austria; g University Clinic of Radiology, Medical University Innsbruck, Innsbruck, Austria; h Institute of Clinical Epidemiology, Tirol Kliniken, Innsbruck, Austria; i Institute of Electrical and Biomedical Engineering, University for Health Sciences, Medical Informatics and Technology (UMIT), Hall in Tirol, Austria

**Keywords:** High sensitivity troponin T, N-terminal pro brain natiuretic peptide, Severe aortic stenosis, Valve replacement, Survival, Risk stratification

## Abstract

Hereby, the supplemental data of the research article “Long-Term Prognostic Value of High-Sensitivity Troponin T added to N-Terminal Pro Brain Natriuretic Peptide Plasma Levels before Valve Replacement for Severe Aortic Stenosis” are presented [1]. It offers enhanced input on the predictive value of these biomarkers considering the influence of the presence of concomitant coronary artery disease (CAD) in various severities as well as an additional cox proportional hazard model on cardiovascular mortality. Furthermore, the receiver operating characteristic (ROC) curves are shown as figures. The material described increases therefore the understanding of the predictive value of these already routinely available biomarkers and reduces the risk of potential bias due to possible confounding factors. It also underlines the urge for a multi-factorial approach in diagnostics to detect the optimal point for referral to valve replacement other than just symptomatic status, an observed reduction in left ventricular ejection fraction or the presence of CAD with the necessity for coronary artery bypass grafting (CABG) [2]. The data of the 3595 patients were gathered retrospectively at a consortium of four university hospital centers in Austria and combined with prospectively collected data on cardiovascular and all-cause mortality.

**Table:** Specifications Table

Subject	Medicine and Dentistry
Specific subject area	Cardiology and Cardiovascular Medicine
Type of data	Table Figure
How data were acquired	Data was retrospectively and prospectively collected. Calculations were conducted with IBM SPSS version 24 (IBM Corporation, Armonk, NY, USA). Graphics were designed by using GraphPad PRISM, version 5 (GraphPad Software, Inc., La Jolla, CA, USA).
Data format	Raw Analyzed
Parameters for data collection	Patients with severe aortic stenosis undergoing either surgical or transcutaneous valve replacement were consecutively enrolled at a consortium of four university hospital centers.
Description of data collection	Data was collected retrospectively at each of the university hospital center either from the local electronic hospital information system or the electronic patient record. Entered data was double checked to reduce the possibility of potential errors. Collected data was then paired with prospective data on cardiovascular and all-cause mortality obtained by “Statistics Austria”, the governmental statistic department.
Data source location	Institution: Medical University Innsbruck City/Town/Region: Innsbruck, Tirol Country: Austria
Data accessibility	With the article
Related research article	Barbieri F, Senoner T, Adukauskaite A, Dobner S, Holfeld J, Semsroth S et al. Long-Term Prognostic Value of High-Sensitivity Troponin T added to N-Terminal Pro Brain Natriuretic Peptide Plasma Levels before Valve Replacement for Severe Aortic Stenosis. Am J Cardiol. 2019; 124(12):1932-1939.

**Table:** 

**Value of the Data** • Why are these data useful? This data helps to identify that concomitant CAD does not reduce the prognostic value of the assessed cardiac biomarkers. • Who can benefit from these data? This data is meant to be hypothesis-generating for clinical researchers as well as for clinicians in daily practice. • How can these data be used for further insights and development of experiments? It may help in clinical decision process. Further values described here may be used for power analysis to estimate the effect on prediction.

## 1 Data

Concomitant significant CAD was present in 1402 (39.0%) patients. Out of those 622 (17.3%) had a one-vessel, 337 (9.4%) a two-vessel, 310 (8.6%) a three-vessel and 133 (3.7%) left-main disease. Coronary revascularisation either via concomitant coronary artery bypass grafting surgery (443, 31.6%) or via percutaneous coronary intervention (410, 29.2%) was performed in 853 (60.8%) patients.

**Fig. 1:**
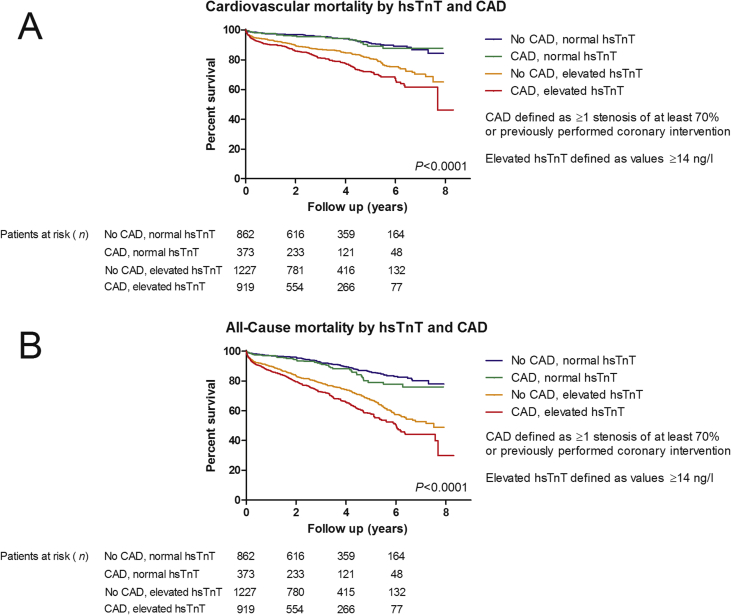
Kaplan-Meier estimates of cardiovascular (A) and all-cause mortality (B) in patients after aortic valve replacement due to severe stenosis, stratified to pre-procedural high sensitivity troponin t plasma levels by using a cut-off point at 14 ng/l and the prevalence of coronary artery disease.

Occurrence of significant CAD separated by high-sensitivity Troponin T (hsTnT) groups is displayed in Table 1. Occurrence of CAD was significantly increased in patients with pathological hsTnT (≥14 ng/l; p < 0.001). For further analysis of the influence of CAD on hsTnT and its prognostic value patients were divided into four groups as following: no significant CAD and normal hsTnT (group 1; n = 862, 25.5%), significant CAD and normal hsTnT (group 2; n = 373, 11.0%), no significant CAD but pathological hsTnT (group 3; n = 1227, 36.3%), significant CAD and pathological hsTnT (group 4, n = 919, 27.2%). Assessment of cardiovascular mortality as well as all-cause mortality by using above mentioned groups is shown in Fig. 1A, B.

**Table 1:** Amount of significantly narrowed coronary arteries separated by hsTnT groups.

No. of coronary arteries narrowed	Normal hsTnT (<14 ng/l)	Pathological hsTnT (≥14 ng/l)
None	862 (69.8%)	1227 (57.2%)
1	181 (14.7%)	403 (18.8%)
2	91 (7.4%)	220 (10.3%)
3	61 (4.9%)	223 (10.4%)
Left Main	40 (3.2%)	73 (3.4%)

**Fig. 2:**
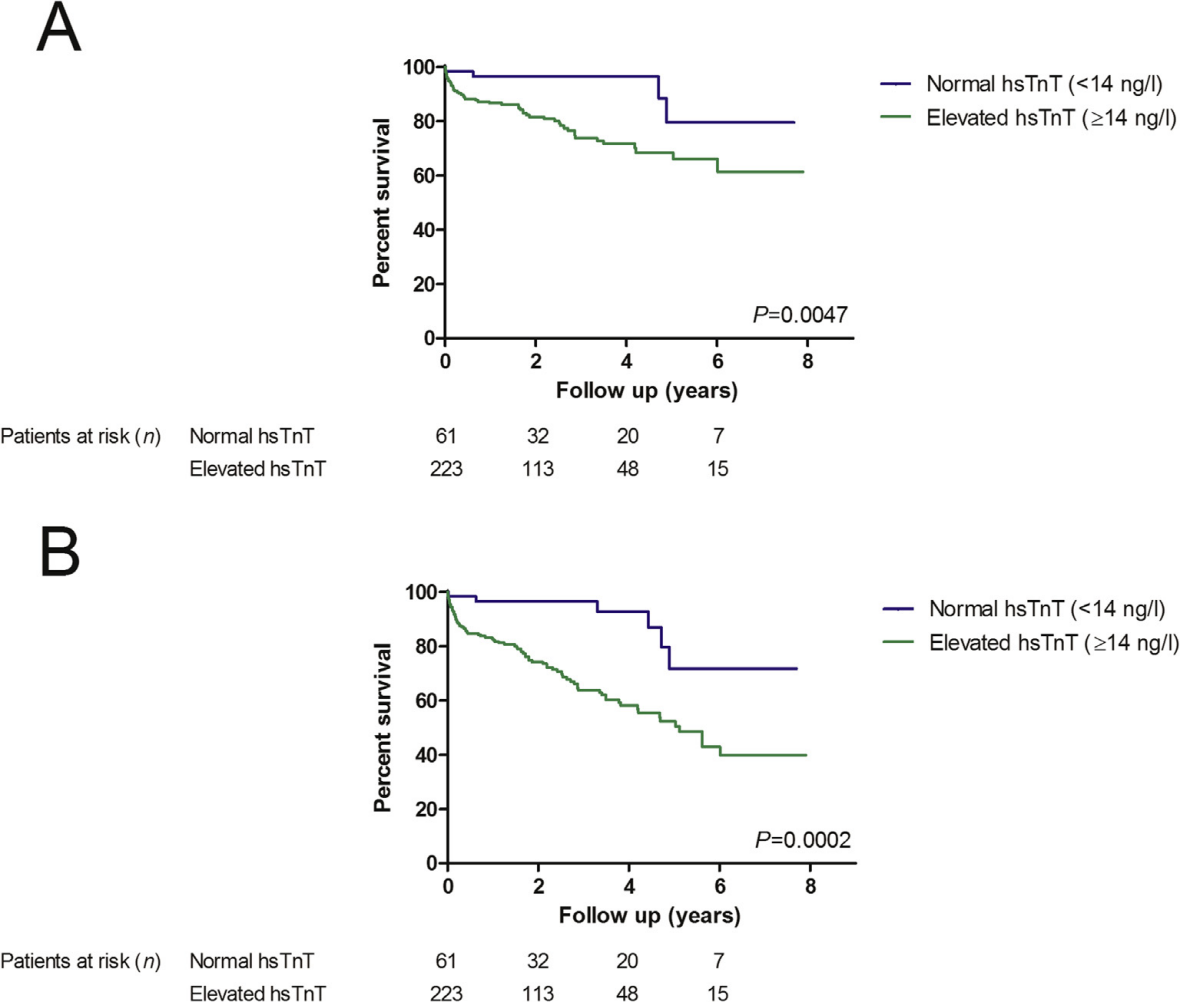
Kaplan-Meier estimates of cardiovascular (A) and all-cause mortality (B) in patients with three-vessel coronary artery disease after aortic valve replacement due to severe stenosis, stratified to pre-procedural high sensitivity troponin t plasma levels by using a cut-off point at 14 ng/l.

**Fig. 3:**
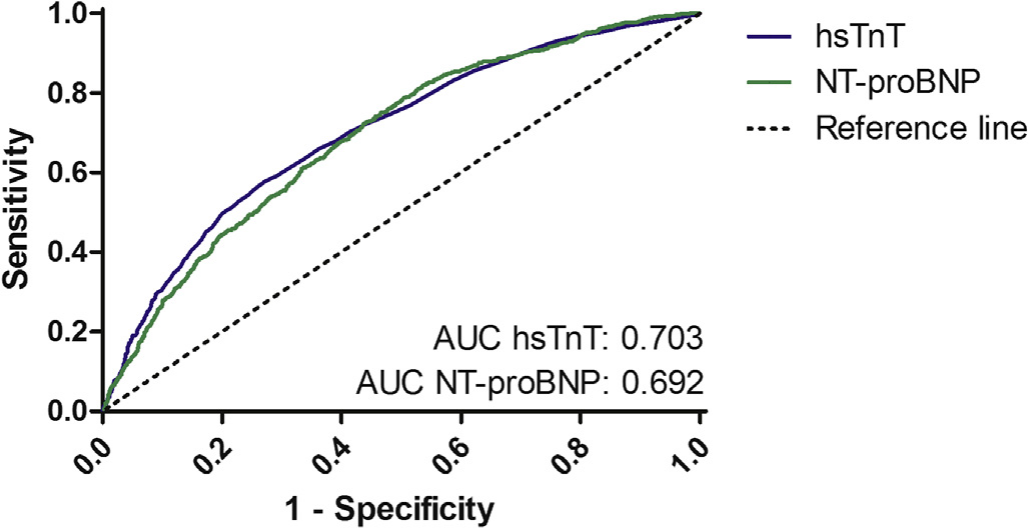
Receiver operating characteristic curve for the evaluation of the predictive value of pre-procedural high sensitivity troponin t and N-terminal pro brain natriuretic peptide plasma levels on all-cause mortality.

Three-vessel disease was found in 284 patients of whom 61 had normal hsTnT and 223 had elevated hsTnT. Kaplan-Meier curves for the prediction of cardiovascular and all-cause mortality by using the hsTnT groups in patients with three-vessel disease are presented in Fig. 2A, B. The ROC curves of N-terminal pro brain natriuretic peptide (NT-proBNP) and hsTnT on all-cause mortality are displayed in Fig. 3. The Youden index, which describes the most sensitive and specific value for the prediction of mortality, was detected to be at 1295 ng/l for NT-proBNP and at 25 ng/l for hsTnT. The cox regression hazard model on cardiovascular mortality is presented in Table 2.

**Table 2:** Multivariate cox regression analysis for cardiovascular mortality.

Variable	HR (95% CI)	*P* value for heterogenity
hsTnT (<14 ng/l as reference)	1.234 (0.940–1.621)	0.130
NT-proBNP (normal range as reference)	1.408 (1.026–1.933)	0.034
LVEF (>50% as reference)		
LVEF (30–50%)	1.087 (0.865–1.365)	0.474
LVEF (<30%)	1.825 (1.265–2.632)	0.001
Age	1.051 (1.032–1.070)	<0.001
CAD	1.188 (0.976–1.445)	0.085
Male gender	0.950 (0.773–1.168)	0.629
Arterial hypertension	1.104 (0.848–1.437)	0.464
Atrial fibrillation	1.813 (1.493–2.201)	<0.001
eGFR	0.991 (0.987–0.996)	<0.001
COPD	1.287 (1.001–1.654)	0.049
Diabetes mellitus	1.115 (0.892–1.393)	0.339
STS-PROM score (<3% as reference)		
3 – <8%	0.987 (0.757–1.287)	0.922
8 – <15%	1.059 (0.678–1.656)	0.801
≥15%	0.991 (0.228–4.301)	0.991
TAVR (SAVR as reference)	1.812 (1.416–2.320)	<0.001

Abbreviations: CAD, coronary artery disease; CI, confidence interval; COPD, chronic obstructive pulmonary disease; eGFR, estimated glomerular filtration rate; HR, hazard ratio; hsTnT, high sensitivity troponin T; LVEF, left ventricular ejection fraction; NT-proBNP, N-terminal pro brain natriuretic peptide; SAVR, surgical aortic valve replacement; STS-PROM score, Society of Thoracic Surgeons predicted risk of mortality score; TAVR, transcatheter aortic valve replacement.

## 2 Experimental design, materials, and methods

Data was acquired locally at each of the contributing hospitals in a retrospective way by using the electronic hospital information system or the electronic patient record. Patients were enrolled consecutively at all participating centers, inclusion and exclusion criteria were described in the main publication [1]. Then, it was paired with prospective data on mortality and cause of death defined by ICD-10 codes. This data was obtained by “Statistics Austria”, the governmental statistic department.

Severitiy of aortic valve stenosis was graded according to current guidelines [2]. The coronary anatomic status was assessed by using coronary angiography in every patient as part of the diagnostic examination prior to valve replacement. Significant CAD was defined as a stenosis of at least 70% or previously performed coronary intervention including CABG. In case of a transcutaneous aortic valve replacement, percutaneous coronary intervention was performed in advance during a separated hospital stay. Revascularisation by coronary artery bypass grafting was conducted as part of the aortic valve replacement surgery.

Chi-square tests were used for the assessment of differences in the prevalence of CAD and hsTnT groups. Analysis of survival was performed by using either the Kaplan-Meier method (univariate) or a cox regression hazard model (multivariate). Calculations were conducted with IBM SPSS version 24 (IBM Corporation, Armonk, NY, USA). Graphics were designed by using GraphPad PRISM, version 5 (GraphPad Software, Inc., La Jolla, CA, USA).
